# Elias George Hall

**Published:** 1942

**Authors:** 


### ELIAS GEORGE HALL, M.B. (Lond.), M.B., Ch.B. (Brist.), M.R.C.S., L.R.C.P.

We regret to record the death of Dr. E. G. Hall which took place on Wednesday, November 18th, at the age of 75.

Dr. Hall was one of our senior Bristol alumni. He entered University College in 1885 and attended the clinical practice of the Bristol Royal Infirmary. He qualified M.R.C.S., L.R.C.P. in 1890 and took the London M.B. degree in 1893. In 1912 he obtained the M.B., Ch.B. degree by examination in the University of Bristol under the regulations laid down for old students of University College.

Dr. Hall held the resident appointment of Junior House Surgeon and Anæsthetist at the B.R.I. When he left the B.R.I. he was appointed to the Bristol Dispensary and worked there for many years. He was also out-patient physician to the Royal Hospital for Women and Children. During the last war he worked as an R.A.M.C. (T.) officer at the Subsidiary War Hospital at the Red Maids' School. He was one of the prime movers in establishing the local volunteers during the last war: a body which corresponds in many ways with the Home Guard of the present war.

Dr. Hall came of a yeoman family in Somerset. He was born at Durdham Lodge, Stoke Bishop, now called The Little Halt. He married, in 1901, Ethel Mary Gilmore, of Barrow Hall, Essex, who survives him and to whom we offer our deep sympathy.

E. G. Hall was a family doctor of the finest type. Kind, sympathetic and unsparing of his own health and strength in the care of his patients.

